# Impact of Preoperative Ineffective Oesophageal Motility on Postoperative Dysphagia Following Nissen Fundoplication: A Systematic Review and Meta-Analysis

**DOI:** 10.7759/cureus.99303

**Published:** 2025-12-15

**Authors:** Varun Arunagiri, Kothai Anbalagan, Sathish Kumar, Abdalazeez Ahmad, Shahriar Md Sadek, Agnes Jenny Cyriac

**Affiliations:** 1 General and Upper GI Surgery, North Devon District Hospital, Barnstaple, GBR; 2 Public Health, The College Merthyr, Merthyr Tydfil, GBR; 3 Surgery, Altnagelvin Area Hospital, Western Trust HSCNI, Derry, GBR; 4 General Surgery, Western Health and Social Care Trust, Londonderry, GBR; 5 Colorectal Surgery, Bangladesh Institute of Health Sciences Hospital, Dhaka, BGD; 6 Family and Community Medicine, St. John's Medical College, Rajiv Gandhi University of Health Sciences, Bangalore, IND

**Keywords:** dysphagia', gerd patients, ineffective esophageal motility, metanalysis research, nissen fundoplication (nf)

## Abstract

Nissen fundoplication (NF) is one of the most common anti-reflux surgeries (ARS) performed worldwide for gastro-oesophageal reflux disease (GERD). There is a theoretical belief that ineffective oesophageal motility (IEM) can cause increased postoperative dysphagia (POD) following NF due to weak and failed peristalsis against a 360-degree wrap. There is scarce evidence to support the hypothesis that NF in pre-existing ineffective oesophageal motility causes increased POD. This study aims to identify the impact of preoperative IEM on POD following NF.

In this study, based on the PRISMA guidelines, we conducted a systematic search for articles published in PubMed, Scopus, EMBASE, as well as in the Cochrane Library, using the search terms "ineffective oesophageal motility", "motility disorder and fundoplication", "Esophageal hypomotility", "Ineffective esophageal motility and GERD" from the inception of these databases to an end date of September 22, 2025. The study participants included patients who had NF for GERD and who had pre-existing IEM. These patients were compared with NF patients with normal motility or non-IEM patients. The outcome measured was POD following NF. The odds ratio and its 95% confidence interval were calculated using the random-effects model. A sensitivity analysis was performed to assess heterogeneity within the study population. For publication bias funnel plots and risk of bias assessment in randomised controlled trials, the Risk of Bias 2 (RoB) is used. For non-randomised studies, the Newcastle-Ottawa Scale (NOS) score is employed.

A total of 10 articles were identified in the search, involving 1473 patients, of whom 1093 had undergone Nissen fundoplication. Among these 1093 patients, 427 had pre-existing IEM. The forest plot in this study suggests that there is no statistical significance in the POD between IEM and non-IEM patients following NF (P = 0.30, Odds ratio = 1.20, overall effect Z = 1.04, df = 5, I^2^ = 0%, 95% CI 0.82, 1.71). There is no statistically significant improvement in POD in patients with IEM compared with preoperative dysphagia following NF in RCTs. In contrast, observational studies show considerable improvement in POD in patients with IEM following NF. The overall effects yielded a p-value = 0.01, OR = 0.40, 95% CI, overall effect Z = 2.54, I^2^ = 73%.

Following NF, POD occurred at similar rates in both IEM and non-IEM patients (P = 0.30, OR = 1.2). NF does not worsen the preoperative dysphagia in patients with IEM and GERD. There can be other factors which worsen postoperative dysphagia. In fact, POD has improved following NF in IEM (P = 0.01, OR = 0.40); however, these results are based primarily on observational studies and on heterogeneous data (I^2^ = 73%).

## Introduction and background

Gastroesophageal reflux disease (GERD) is among the most common upper GI diseases worldwide [1,2]. Gastroesophageal reflux is a normal physiological process. This physiological process can become pathological when it impairs quality of life and becomes a disease when symptoms worsen [3, 4]. The treatment of GERD ranges from lifestyle modification and medical management with proton pump inhibitors to surgical management [5,6]. There is a range of surgical operations available to alleviate the symptoms of GERD. Nissen fundoplication (NF) is one of the common operations performed to treat GERD [7]. Oesophageal motility disorders, on the other hand, have received increasing attention in recent years, driven by the growing use of high-resolution manometry (HRM) [8]. Both GERD and oesophageal motility disorders share common symptoms such as reflux, dysphagia, retrosternal chest pain, and heartburn [9]. The range of oesophageal motility disorders includes and not limited to ineffective oesophageal motility (IEM) and severe forms, such as achalasia cardia and hypercontractile oesophageal motility disorder. IEM is a form of motility disorder where there is weak or failed peristalsis. The definition of IEM has changed, and there has been an overdiagnosis of IEM. The reasons for IEM could include oesophageal inflammation or stricture. GERD causes inflammation of the distal oesophagus, which may explain the associated IEM.

NF has yielded better results in recent years for treating GERD, with long-term symptom relief of 80-95% [10, 11]. The downside of NF is postoperative complications such as postoperative dysphagia (POD), wrap migration, and recurrence of symptoms. To minimise postoperative complications, a comprehensive preoperative evaluation is conducted, including a barium swallow, 24-hour pH monitoring, and high-resolution manometry (HRM) [12]. One of the dreadful preoperative findings is pre-existing oesophageal motility disorders. A pre-existing oesophageal motility disorder can alter the management of GERD [13]. In recent times, HRM has gained popularity as part of preoperative investigations for NF to identify pre-existing oesophageal motility disorders and minimise postoperative complications in fundoplication. NF in pre-existing oesophageal motility disorders, such as achalasia cardia, diffuse oesophageal spasm, and contractile dysfunction, will be a disaster. However, GERD has been diagnosed with IEM in many patients, which accounts for 49 to 53% [14]. In patients diagnosed with GERD and IEM, a partial fundoplication, such as a Toupet Fundoplication (TF) or Dor Fundoplication (DF), is preferred over NF because it has fewer side effects, such as POD. However, the outcomes of GERD are the same in both techniques [15,16].

NF provides a 360° wrap around the gastro-oesophageal junction for reflux disease. IEM or failed oesophageal motility can cause more dysphagia with the 360° wrap [17]. A tight NF, by its very nature, can cause POD. There is limited evidence available in the literature to suggest that all NF in patients with IEM worsens the pre-existing dysphagia. In contrast, many studies indicate that there is a better outcome in patients with preoperative dysphagia. There is no systematic review and meta-analysis to create strong evidence either in favour of NF or against NF in IEM patients with preoperative dysphagia. This study aims to evaluate the impact of preoperative IEM in patients undergoing NF. The primary objective is to identify the number of patients with postoperative dysphagia following NF with IEM. The hypothesis considered in this study is that NF causes worsening preoperative dysphagia in patients who have preoperative dysphagia and IEM. The primary outcome measured is postoperative dysphagia in patients with IEM following NF, compared to patients with normal motility. Also, the authors analysed the preoperative dysphagia and postoperative dysphagia in patients with IEM.

## Review

Materials & methods

In accordance with the Preferred Reporting Items for Systematic Reviews and Meta-Analyses (PRISMA 2020) statement, a comprehensive systematic review and meta-analysis were performed [18]. Given that this systematic review incorporates all available evidence from published articles online, and as the evidence is publicly available for review, institutional review board approval is not required.

Search strategy and literature screening

**Figure 1:**
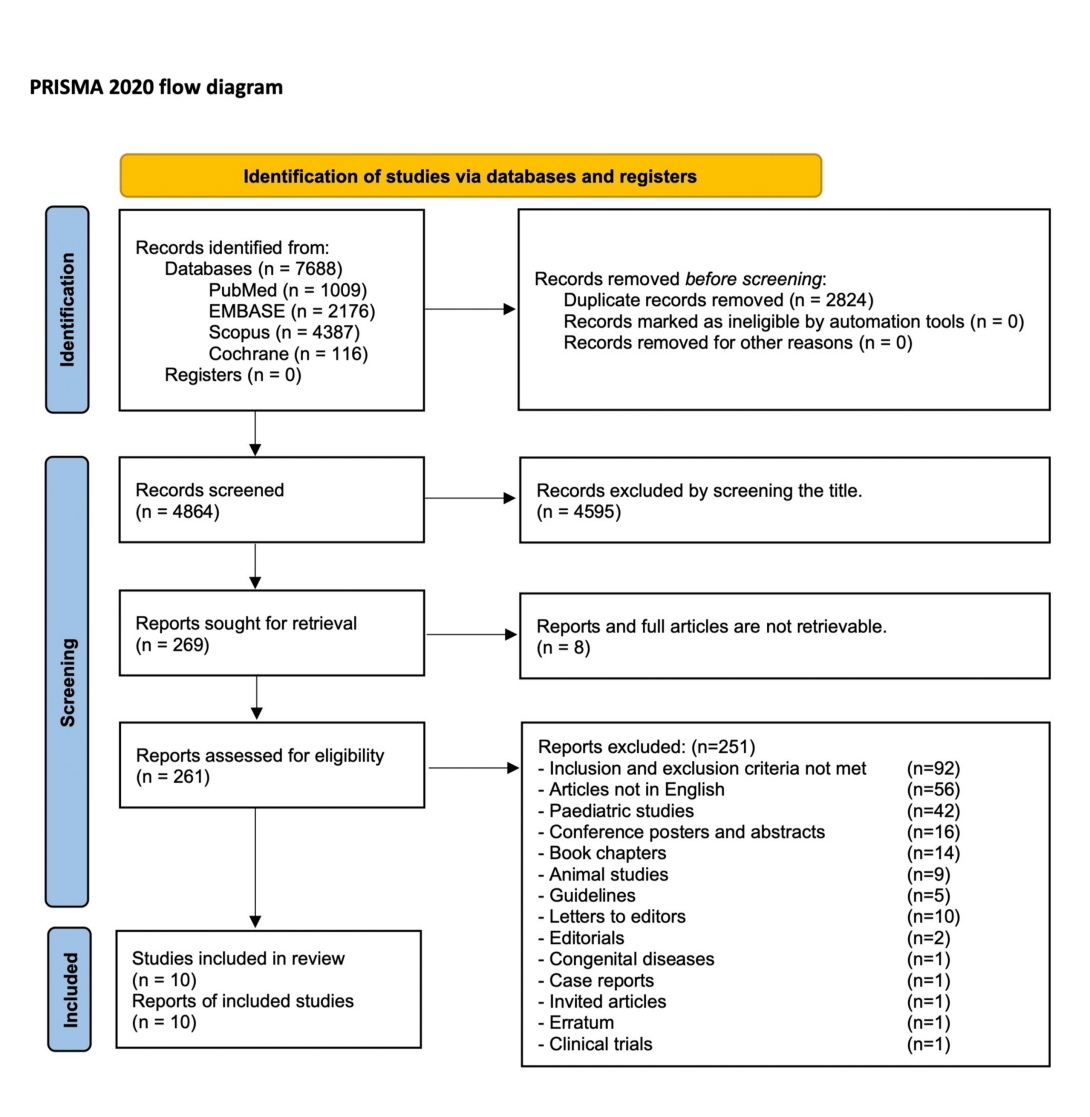
PRISMA flow diagram.

A systematic electronic search was conducted in PubMed, Embase, Scopus, Ovid full-text journals, and the Cochrane Library, from the inception of the databases to September 22, 2025, by the authors, VA and KA, separately. The literature search was conducted again on November 21, 2025, to identify any new articles. In the databases PubMed, EMBASE, Scopus, the authors used the terms "Ineffective Oesophageal motility", "Oesophageal hypomotility" in all fields, "Ineffective Oesophageal motility AND GERD", and "Motility disorder AND fundoplication" using the title and abstract filters. In the Cochrane review, the same terms were used in the filter, title, abstract, and keyword searches. All the articles published in any language were searched to include in the study. However, only English-language articles were identified. The search terms used to screen article titles were “ineffective oesophageal motility”, “Nissen fundoplication”, and “fundoplication”. All articles identified using the search terminology from different databases were submitted to rayyan.ai in Excel (CSV format) for further review and data extraction. In total, 7688 articles were identified from all the databases: PubMed-1009, EMBASE-2176, Scopus-4387, and Cochrane-116. A total of 2824 duplicates were removed using rayyan.ai, and the remaining 4864 records were screened manually by both authors (VA and KA) with blinds on. After the title screen, 269 articles were identified for title and abstract screening. Eight full articles were not retractable. Two hundred and sixty-one full articles were screened for eligibility for inclusion and exclusion criteria. Ten articles were eventually identified for inclusion in review and analysis. When there is disagreement over decisions between the reviewers (VA and KA), the dispute is resolved after discussion with a third reviewer (SM). The PRISMA flow diagram was then created (Figure 1).

Study selection with inclusion and exclusion criteria

All studies, including randomised controlled trials, retrospective case-control studies, and prospective studies, were included in the analysis. Inclusion criteria are patients who were diagnosed with GERD, who have had NF, and who had a preoperative diagnosis of IEM and normal motility. The exclusion criteria are the patients who had systemic diseases like Raynaud's, scleroderma, systemic sclerosis, lung transplantation, congenital diseases, who had functional Luminal impedance manometry (FLIP) either intraoperatively or postoperatively, paediatric population, animal studies, patients with other oesophageal motility disorders, including achalasia cardia and hypercontractile oesophagus, and cases without a clear definition of ineffective oesophageal motility. Patients who had fundoplication procedures other than NF or LINX were excluded. Studies published as conference abstracts, letters to the editor, narrative reviews, and articles without complete data were excluded.

Definitions considered in the study

The definition of the IEM has changed over the decades. For the study and to include all patients with IEM in the literature, the following definition is used. The definition of IEM, which was conventionally used during the 1990s when conventional manometry was employed, differed [19]. IEM was diagnosed with conventional manometry when there is a presence of >30% ineffective oesophageal motility. In recent years, HRM has been utilised with all versions of the Chicago Classification (CC), including v1.0, v2.0, v3.0, and v4.0. With the advent of HRM, the definition has become more stringent, leading to overdiagnosis of IEM. IEM is defined as weak oesophageal contractions, with ≥30% of ineffective swallows, distal contractile integral (DCI) <450 mmHg/s/cm, and at least 50% of failed peristalsis. This definition encompasses all patients in the literature who were diagnosed with IEM. The present CC4.0 defines IEM as >70% ineffective swallow or ≥50% failed peristalsis, and DCI <100 mmHg/s/cm with a normal Integrated Relaxation Pressure (IRP) [20].

NF is defined as a 360-degree wrap around the gastroesophageal junction. Robotic, laparoscopic and open NF is considered for the study. The wrap length is not accounted for in the study. Some studies consider the use of a bougie, which is not considered. A floppy NF is regarded as a part of the definition of NF. According to the Lyons criteria, GERD is defined as chronic symptoms or mucosal damage incurred by the abnormal reflux of gastric contents into the oesophagus and beyond.

POD is defined as patients presenting to the clinic with symptomatic dysphagia or difficulty in swallowing. Early POD (up to three months) is not included in the study analysis. Symptomatic POD during the follow-up period of six months or more is regarded as POD.

Data extraction

Full articles were screened for the data extraction. Data extracted from the study demographics included the total study population (n), age (mean or median age group with range), and sex of the study population. The Body Mass Index (BMI), if available, was also extracted. Other data considered were the coexistence of hiatus hernia (HH). The total number of patients who underwent NF in the IEM and non-IEM categories was extracted. Also, to assess the outcome, preoperative dysphagia in both patients with IEM and non-IEM following NF was retrieved. Studies were searched for data in tables, charts, and graphs to retrieve the information. When direct values are not available, percentages are converted to whole numbers. Search for statements in the studies regarding the preoperative dysphagia and POD were analysed to extract the data. The NF-only data extraction from the multi-arm trials by Zornig et al. and Strate et al. was based solely on the tables and graphs provided in the articles. These studies feature clear tables that facilitate the extraction of events and controls.

Statistical analysis

Statistical analysis was done using RevMan 5.0 (The Cochrane Collaboration, London, England). Dichotomous data were used to analyse the outcomes. As there are four RCTs and six observational studies, the analysis was done in two groups, and the total effect was calculated. RCTs were analysed separately, and observational studies were analysed independently; the total outcome was then measured. Data heterogeneity was initially assessed through visual inspection of the forest plot and subsequently with I2 statistics. Data heterogeneity is considered significant when p < 0.10 or I2 > 50%. Data heterogeneity is regarded as a problem when studies include both RCTs and observational studies. When there is no heterogeneity, fixed effects are used to obtain the results. The random effects model is used to analyse study results when data are heterogeneous. When random effects are used to analyse the results, the Confidence Interval (CI) is calculated using the Wald-type method, and heterogeneity (Tau^2^) is estimated using the Restricted Maximum Likelihood (REML) method. As the prevalence is <10% and the study includes both RCTs and retrospective case-control studies, the Odds ratio is used for analysis. Moreover, because the variables were dichotomous, Odds ratios with 95% CIs were used to report the results. For continuous data, the mean difference or the mean with Standard deviation with 95% CI was used to report the results. A funnel plot was used to assess publication bias. Risk of Bias (RoB) is estimated using the RoB 2 for RCTs [21] and the Newcastle-Ottawa Scale (NOS) for non-randomised studies [22].

Results

Ten articles were included in the qualitative and quantitative analysis [23-32]. There were four RCTs [23-26] and six observational studies [27-32]. After the PRISMA flow chart was completed, data extraction from all the articles was performed and entered into RevMan 5.0. The study events were then analysed.

Study demographics

A total of 1,473 patients were identified across all 10 studies. Eight hundred sixty-three had normal motility, and 610 patients had IEM. A total of 1093 patients had NF, of whom 427 had IEM, and 666 had non-IEM or normal motility. Table 1 describes the study details. The RCT studies [23, 25, 26], which had both NF and TF arms in their primary study, extracted data from the NF arm only, and ensured that appropriate data were extracted from the study tables and graphs.

**Table 1:** Studies included in the analysis. NF: Nissen Fundoplication, IEM: Ineffective esophageal Motility. Zornig et al. [23] and Strate et al. [25] have different inclusion periods and follow-up. Though they appear similar, both studies are included due to different study periods and follow-up periods.

Study/Author	Year	Country	Journal	Study design	Total Number of patients in the study (n)	IEM (n)	Normal Motility (n)	Nissen's Fundoplication (n)	NF in IEM (n)	NF in Normal motility (n)
Zornig et al. [23]	2002	Germany	Surgical Endoscopy	RCT	200	100	100	100	50	50
Fibbe et al. [24]	2001	Germany	Gastroenterology	RCT	200	100	100	100	50	50
Strate et al. [25]	2008	Germany	Surgical Endoscopy	RCT	200	100	100	100	50	50
Booth et al. 2008 RCT [26]	2008	UK	British Journal of Surgery	RCT	127	52	75	64	26	38
Addo et al. [27]	2020	USA	Surgical Endoscopy	Retrospective Case Control	203	44	159	186	37	149
Booth et al. [28]	2002	UK	Diseases of the Oesophagus	Retrospective Case Control	117	35	82	117	35	82
Nikolic et al. [29]	2019	Austria	World Journal of Surgery	Retrospective Case-matched study	144	72	72	144	72	72
Ravi et al. [30]	2005	Ireland	The American Journal of Surgery	Retrospective Case Control	98	38	60	98	38	60
Munitiz et al. [31]	2004	Spain	British Journal of Surgery	Retrospective Case-Control	93	41	52	93	41	52
Tsereteli et al. [32]	2009	USA	Surgical Endoscopy	Retrospective Case Control	91	28	63	91	28	63
				Total	1473	610	863	1093	427	666

The age distribution of the population, BMI, and other characteristics, such as hiatus hernia, are mentioned in Table 2. There is no statistical significance in the covariates in the studies. Fibbe et al. have not included the covariates age and BMI [24]. Booth et al. (2008) reported that, in their RCT, the mean age of the study population was calculated as a single category for both IEM and non-IEM patients [26]. No data are available for BMI. However, the study has used the population's mean weight. The IEM definition used in each study is listed in Table 3.

**Table 2:** Study demographics GERD: Gastro Oesophageal Reflux Disease, NF: Nissen Fundoplication, IEM: Ineffective Oesophageal Motility, NA: Not available, BMI: Body Mass Index, HH: Hiatus hernia; Values in parentheses are range.

Study/Author	Indication for Fundoplication	Age Mean/Median	P-value from the studies for age groups in IEM and non-IEM	BMI mean (Range)	P-value from the studies for BMI in IEM and non-IEM groups	Others
Zornig et al. [23]	GERD	56 (20-80)		26.4 (18.9-40.4)		
Fibbe et al. [24]	GERD	NA		NA		
Strate et al. [25]	GERD	56 (20–80)		26.4 (18.9-40.4)		
Booth et al., 2008 RCT [26]	GERD	45.3 (21–86)		NA		Mean weight 81.6 (55–103) kg
Addo et al. [27]	GERD	IEM 58.1 ± 15.3	p = 0.062	IEM 27.4 ± 4.1	p = 0.288	
		Non-IEM 62.2 ± 12		Non-IEM 28.2 ± 4.9		
Booth et al. [28]	GERD	IEM 43.9				Mean Weight 75.9 kg
		Non-IEM 43.6				
Nikolic et al. [29]	GERD	Median IEM 55	p = 0.862	Median 27	p = 0.716	HH 2 cm for both IEM and non-IEM
		Median non-IEM 55				
Ravi et al. [30]	GERD	Median 45 (16 to 74)				Median weight 73.2 kg
Munitiz et al. [31]	GERD	IEM 48 (20–74)		NA		
		Non-IEM 46 (16–78)				
Tsereteli et al. [32]	GERD	IEM 53 (30–87)		NA		HH with IEM 6
		Non-IEM 35 (22–80)				HH with Non-IEM 7

**Table 3:** Definition of the ineffective oesophageal motility in all studies.

Study/Author	IEM definition as per the study
Zornig et al. [23]	As a mean contraction amplitude of <40 mmHg and/or failed primary peristalsis of 10 wet swallows in >40%.
Fibbe et al. [24]	Primary peristalsis of 40% or less and/or mean distal oesophageal pressure of less than 40 mmHg.
Strate et al. [25]	Mean contraction amplitude less than 40 mmHg and/or failed primary peristalsis of 10 wet swallows in more than 40%.
Booth et al. 2008 RCT [26]	30% or more of the waves were low in amplitude (less than 30 mmHg in at least one of the two distal sensors) or non-propagated oesophageal body motility was classified as ineffective.
Addo et al. [27]	≥50% ineffective peristaltic sequences (distal contractile integral (DCI) <450 mmHg/s/cm in at least 50% of ten liquid swallows in the presence of normal lower oesophagal sphincter relaxation).
Booth et al. [28]	If 30% or more of waves were low in amplitude (less than 30 mmHg in at least one of the two distal sensors) or non-propagated, oesophageal body motility was classified as ineffective.
Nikolic et al. [29]	An ineffective swallow is characterised by a DCI 450 mmHg/s/cm. IEM is diagnosed if ≥50% of 10 swallows is ineffective.
Ravi et al. [30]	Evidence of hypocontraction in the distal oesophagus with at least 30% of wet swallows exhibiting any combination of the following abnormalities: distal oesophageal peristaltic wave amplitude 30 mmHg, simultaneous contractions with amplitudes 30 mmHg, failed peristalsis in which the peristaltic wave did not traverse the entire length of the distal oesophagus, or absent peristalsis.
Munitiz et al. [31]	50% or more of wet swallows associated with low-amplitude contractions (less than 30 mmHg) in the distal third of the oesophagus or a proportion of simultaneous waves of 30% or more during a standard manometric study.
Tsereteli et al. [32]	Oesophageal body motility is considered to be abnormal if ≥30% of wet swallows exhibited any combination of the following abnormalities: distal esophageal peristaltic wave amplitude <30 mmHg, simultaneous contractions with amplitudes <30 mmHg, failed peristalsis in which the peristaltic wave did not transverse the entire length of the distal esophagus or absent peristalsis.

Outcome results

Postoperative Dysphagia in IEM and Non-IEM Patients

**Figure 2:**
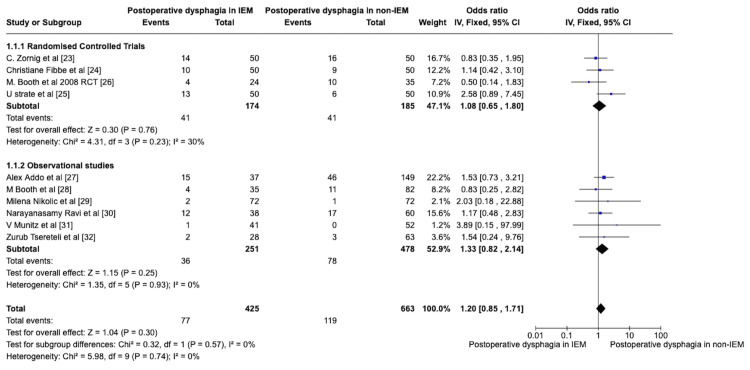
Forest plot comparing the POD in IEM and non-IEM patients following NF POD: Postoperative Dysphagia, IEM: Ineffective Esophageal Motility, NF: Nissen Fundoplication

All the studies were analysed for the number of patients who had preoperative dysphagia in the IEM and non-IEM groups. The patient who had undergone fundoplication other than the NF was excluded, and care was taken to exclude these patients from the outcome analysis. Early POD was not included in the study. POD after six months or more of follow-up was considered an experimental event. Outcome analysis was done to compare postoperative dysphagia in IEM patients who had NF as experimental events and was compared to postoperative dysphagia in non-IEM patients who had NF as a control. The data were entered into RevMan 5.0, and the forest plot was analysed (Figure 2). There was no statistical significance between patients with POD in IEM and non-IEM after NF, as the p-value was greater than 0.10 and I2 was 0%. As the data were not heterogeneous, a fixed-effect model was used to analyse the effects. Both RCT and retrospective studies showed no statistical difference in the postoperative dysphagia in IEM and non-IEM patients. RCT studies comparing the POD in IEM and non-IEM yielded an overall effect size of Z = 0.30 (p-value = 0.76), OR = 1.2, with heterogeneity (*Chi*^2^ = 4.31, df = 3, P = 0.23; *I^2^* = 30%). Retrospective studies yielded the same non-significance in the post-operative dysphagia (overall effect of Z = 1.15 with a p-value of 0.25 with *Chi*^2^ = 1.35, df=5 (P=0.93; I^2^=0%). The overall study yielded a p-value of 0.30, OR = 1.20 with *Chi^2^* = 5.98, df=9, P=0.74; I^2^=0%. There is no statistical significance in POD between patients with IEM and non-IEM following NF, although the OR is 1.20.

Preoperative and Postoperative Dysphagia in the IEM Patients

**Figure 3:**
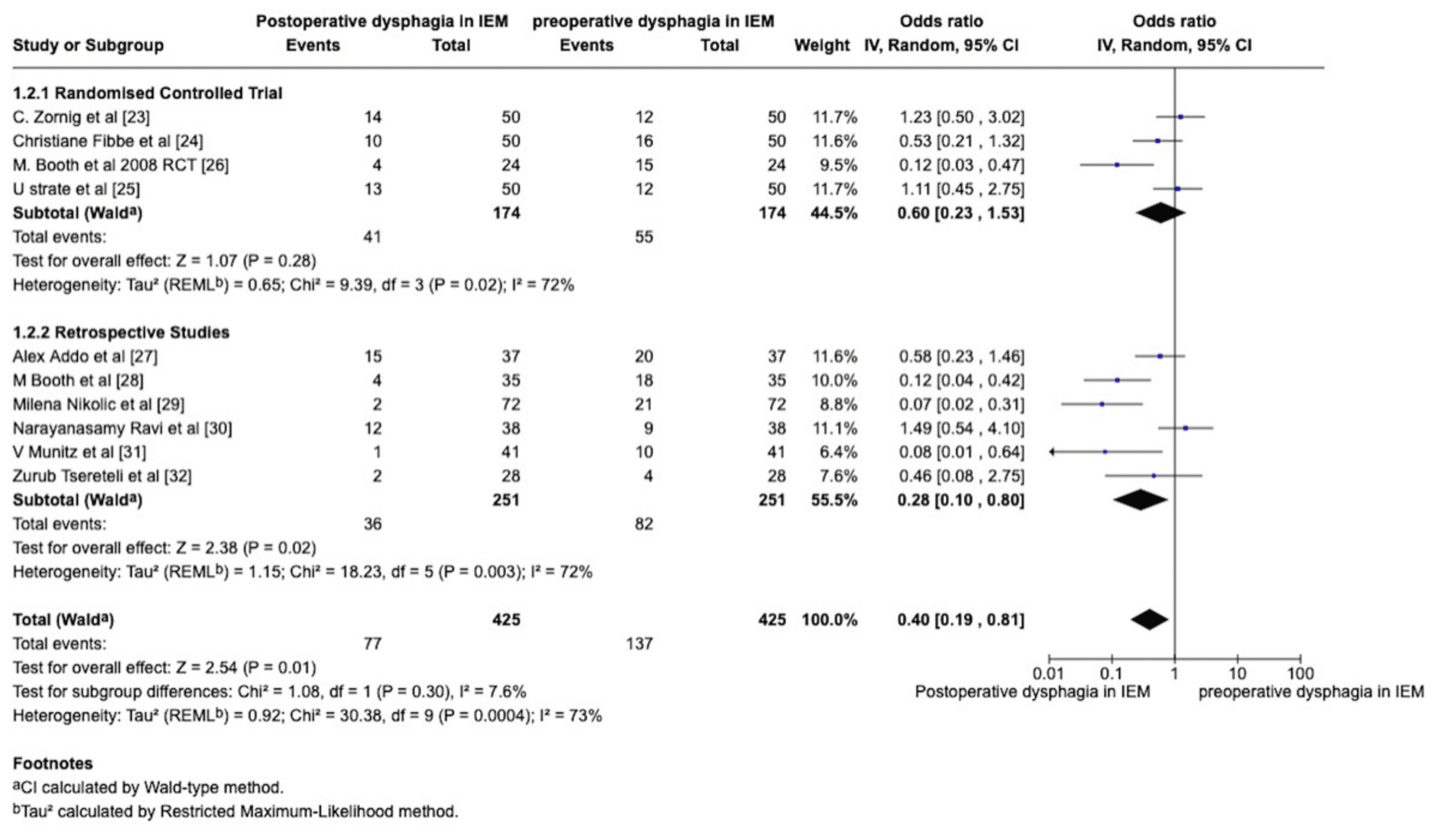
Forest plot comparing the POD and preoperative dysphagia in IEM patients following NF POD: Postoperative Dysphagia, IEM: Ineffective Esophageal Motility, NF: Nissen Fundoplication

The authors compared the preoperative dysphagia and the POD in IEM patients. This comparison was conducted to analyse the statistical significance of the preoperative and postoperative outcomes of POD in patients who underwent NF with a pre-existing dysphagia in IEM patients. Care was taken to exclude all the new-onset or de Novo dysphagia patients after NF. Experimental events were considered to be POD with a control group, considering the preoperative dysphagia in patients in the control group. The forest plot analysis was conducted and is shown in Figure 3. The data heterogeneity exceeded 50%, so a random-effect model was used for statistical analysis.

Analysis of preoperative and postoperative outcomes in both RCT and retrospective studies showed statistical significance. There was a statistical difference between RCT studies and observational studies. RCT studies for preoperative and POD yielded an overall effect of Z = 1.07 with a p-value of 0.23, OR = 0.60 with the Random effects Model Tau² (REML^b^)=0.65; *Chi*^2^= 9.39, df=3, P=0.02; I^2^=0%. In contrast, retrospective studies yielded different statistical results. The overall effects of retrospective studies showed a statistical significance, implying that POD improved in IEM patients following NF (overall effect of Z = 2.38 with a p-value of 0.02 with Random Model Tau² (REMLb)=1.15; *Chi*^2^ = 18.23, df=5 (P=0.003; I^2^=72%)). The Wald test was used to calculate the total effect (Z = 2.54). The overall study yielded a p-value of 0.01, OR = 0.40 with Random effects Model Tau² (REML^b^)=0.92; *Chi*^2^ = 30.38, df=9, P=0.0004; I^2^=073%. The retrospective observational studies obviously influence the overall results.

Outcome analysis of studies based on conventional manometry and HRM using the Chicago Classification 3.0

**Figure 4:**
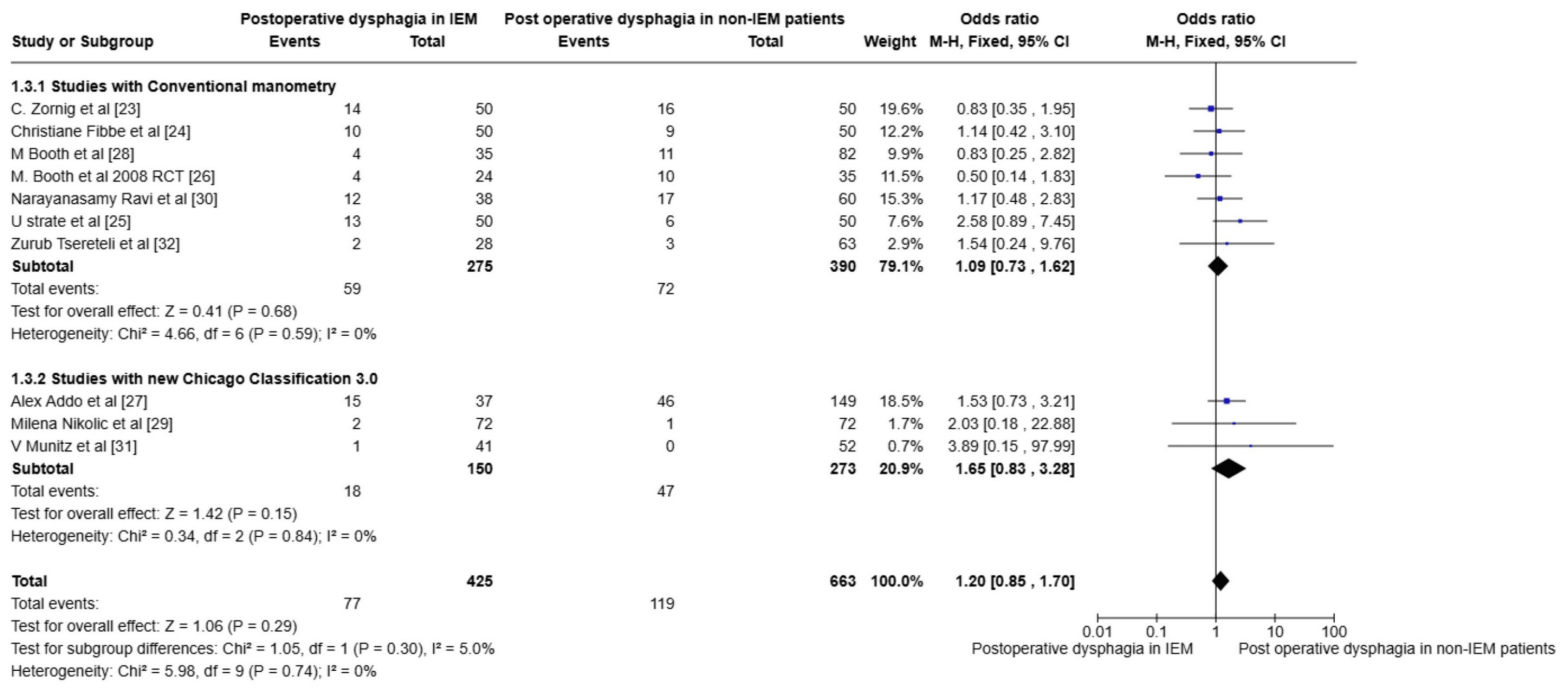
Forest plot categorising the studies based on conventional manometry and HRM analysing the POD in IEM and non-IEM patients HRM: High-resolution manometry, IEM: Ineffective esophageal motility. None of the studies used the Chicago Classification 4.0.

**Figure 5:**
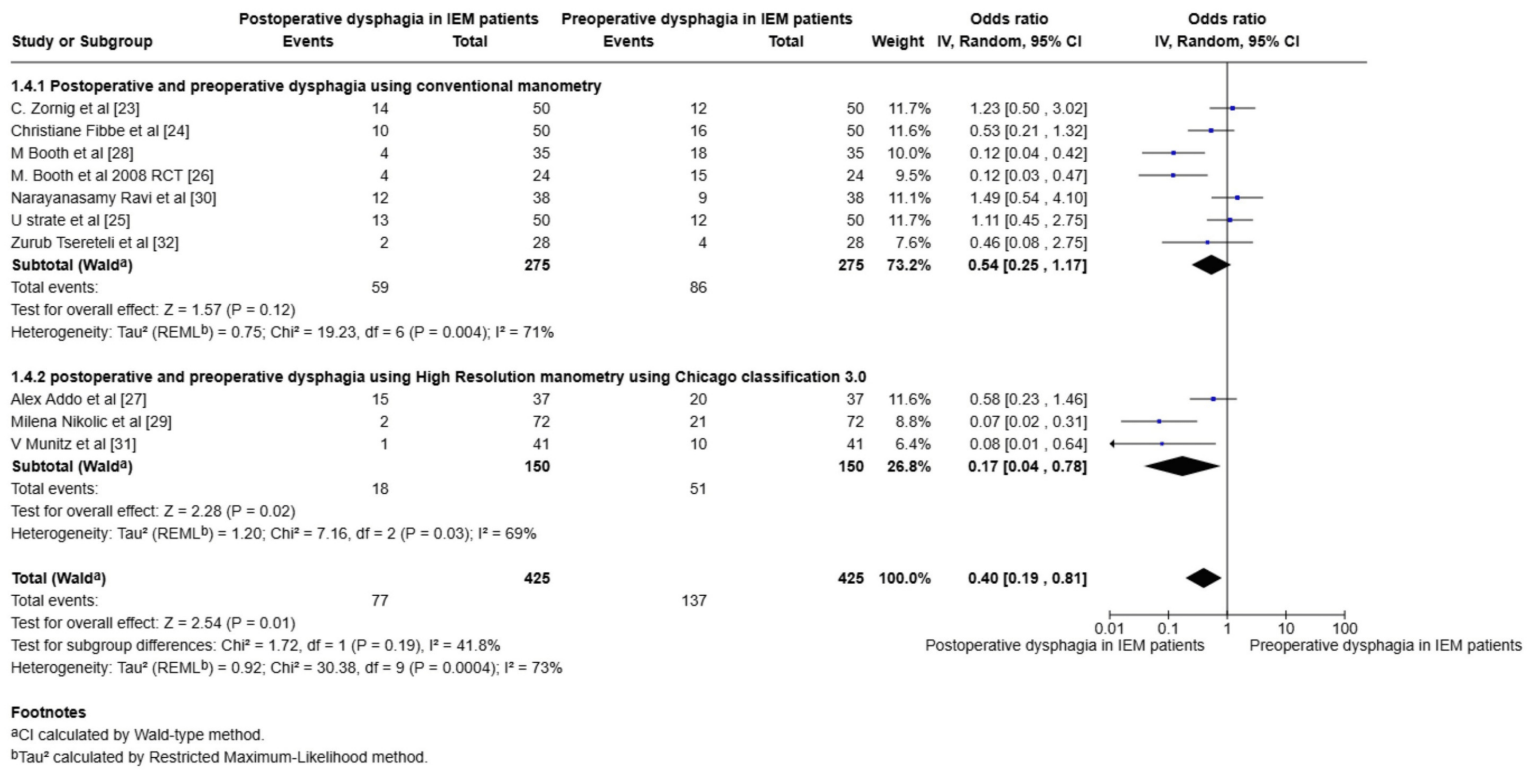
Forest plot analysis after categorising the studies based on conventional manometry and HRM and analysis of preoperative and POD in IEM following NF HRM: High-resolution manometry, IEM: Ineffective esophageal motility, NF: Nissen Fundoplication

The outcomes included heterogeneity due to the use of conventional manometry and HRM to define the IEM. This allowed us to do a subgroup analysis categorising the studies into two groups. Therefore, the studies were classified into two groups: those that used conventional manometry and those that used HRM, according to the Chicago Classification 3.0. A forest plot was created to determine the significance of the outcome. Figure 4 illustrates the forest plot for studies using conventional manometry, with a subgroup analysis of those that used HRM for POD in IEM and non-IEM patients. There is no statistical significance using a fixed model, with a p-value of 0.28 and I2 = 0%. Figure 5 illustrates the forest plot comparing POD and preoperative dysphagia in IEM, with a subgroup analysis of studies that included HRM as the outcome. The results showed high heterogeneity of the data, with an I2 value of 73%. So we used a random effects model, and the results yielded a p-value of 0.0004. This suggests that the POD improved in IEM patients following NF.

Reflux outcomes

Of the 10 studies included in the analysis, the studies varied in their measures of reflux outcomes. The reflux outcomes were measured as Reflux Symptom Index (RSI), Gastroesophageal Reflux Disease Health-Related QOL (GERD-HRQL), postoperative manometry assessment, POD score, Visick score, postoperative dysphagia, and postoperative 24-hour pH monitoring and manometry. There was no statistical significance in any of the outcomes between the normal motility group and the IEM patients. All the patients had reasonable control of reflux symptoms. There was evidence of de Novo dysphagia or new onset dysphagia in both IEM and non-IEM patients following NF.

Risk of Bias (RoB)

**Figure 6:**
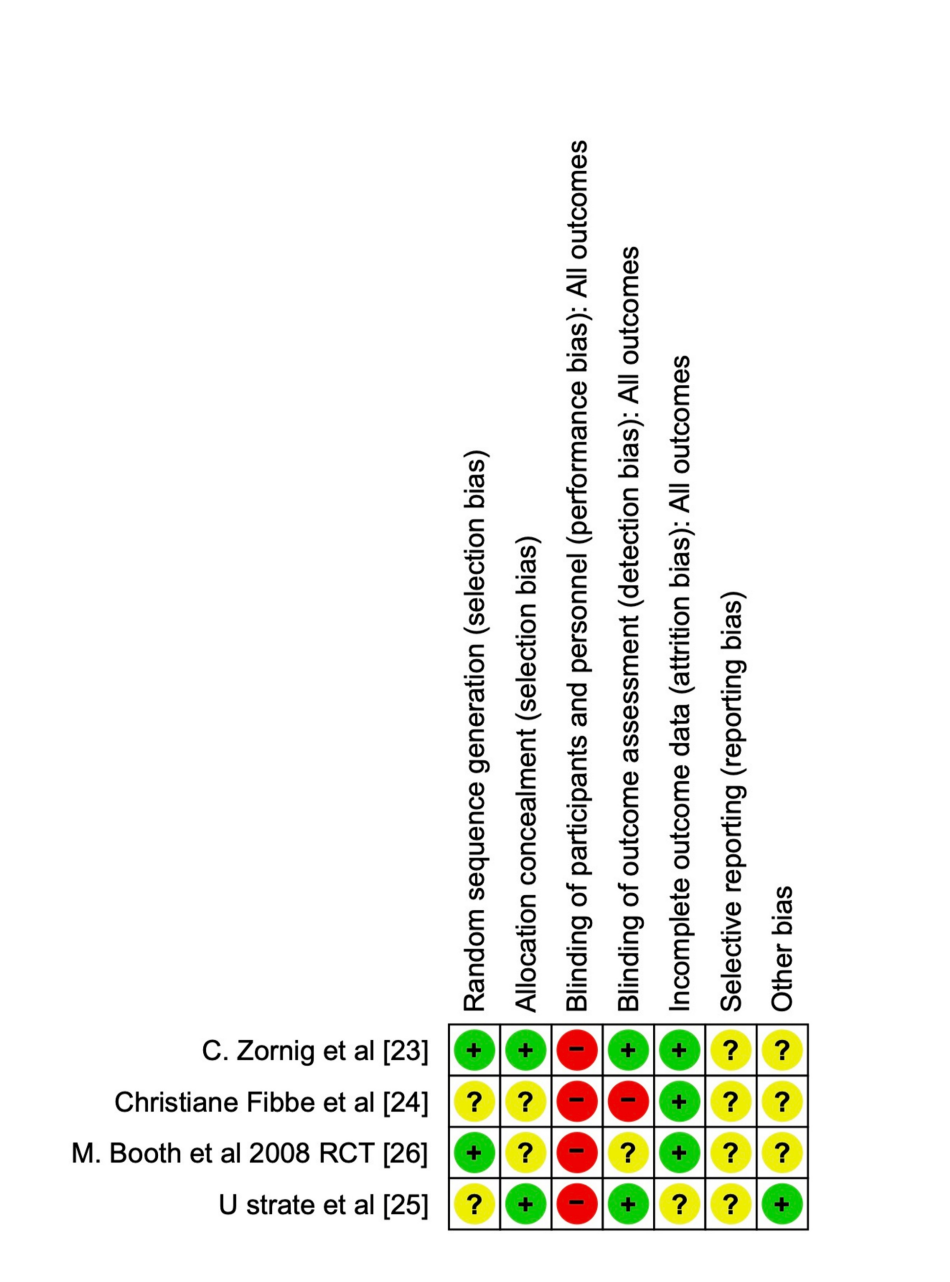
Risk of Bias Summary Graph Green represents -* Low risk of Bias*, Yellow represents - *Unclear risk of Bias*, Red represents - *High risk of bias*

**Figure 7:**
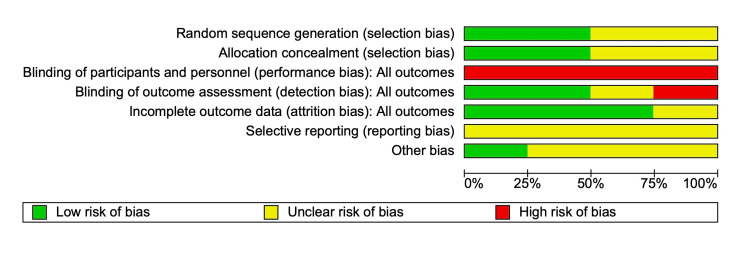
Risk of bias graph for the randomised controlled trial studies Bias graph for the randomised control trials studies [23-26]. Green represents *Low risk of bias*, Yellow represents* unclear risk of bias*, and red represents *High risk of Bias.*

**Figure 8:**
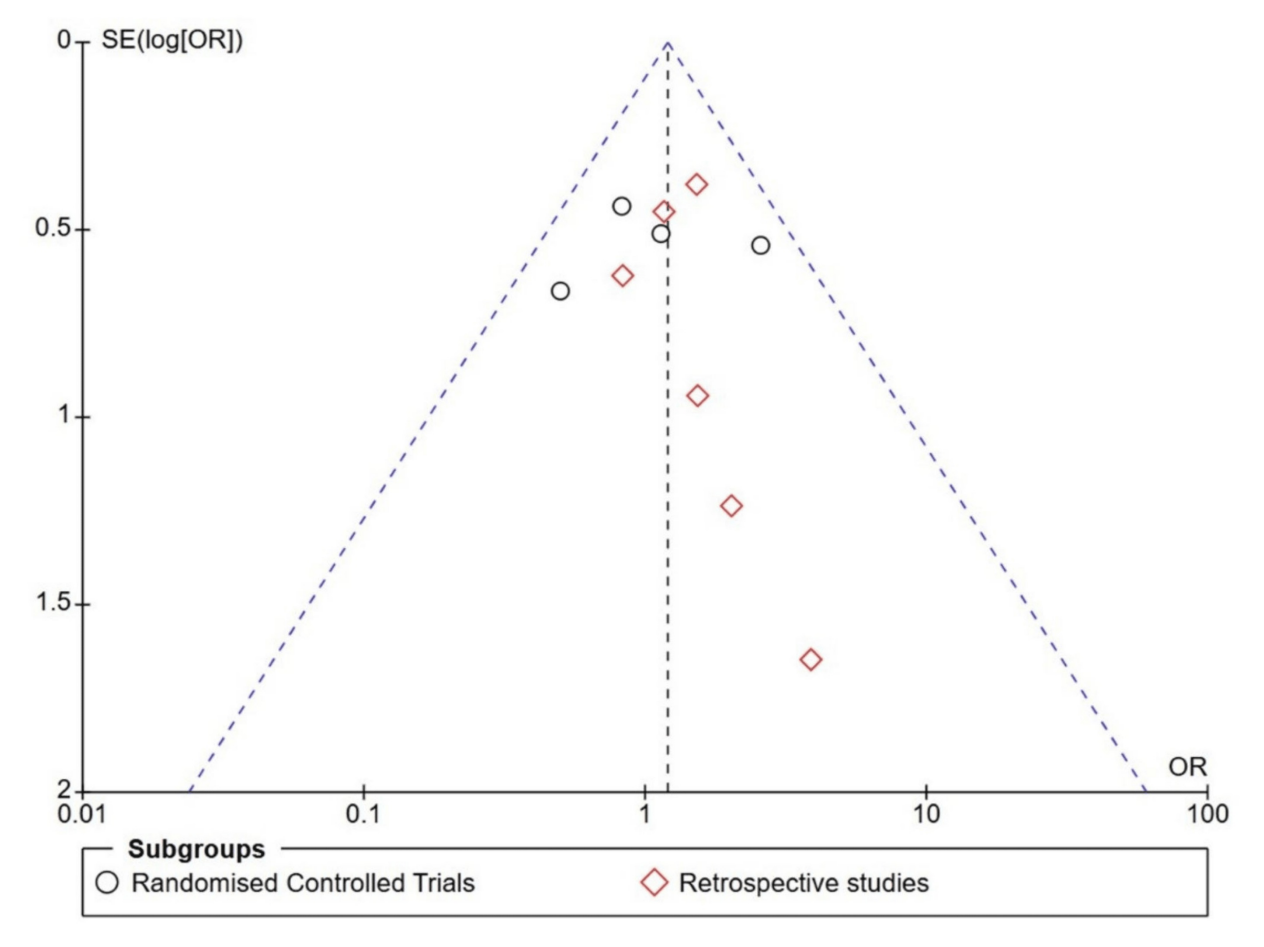
Funnel plot for the publication bias considering the analysis of POD in IEM and non-IEM patients following NF. Black circles represent RCT studies [23-26]. Red diamonds represent observational studies [27-32].

**Figure 9:**
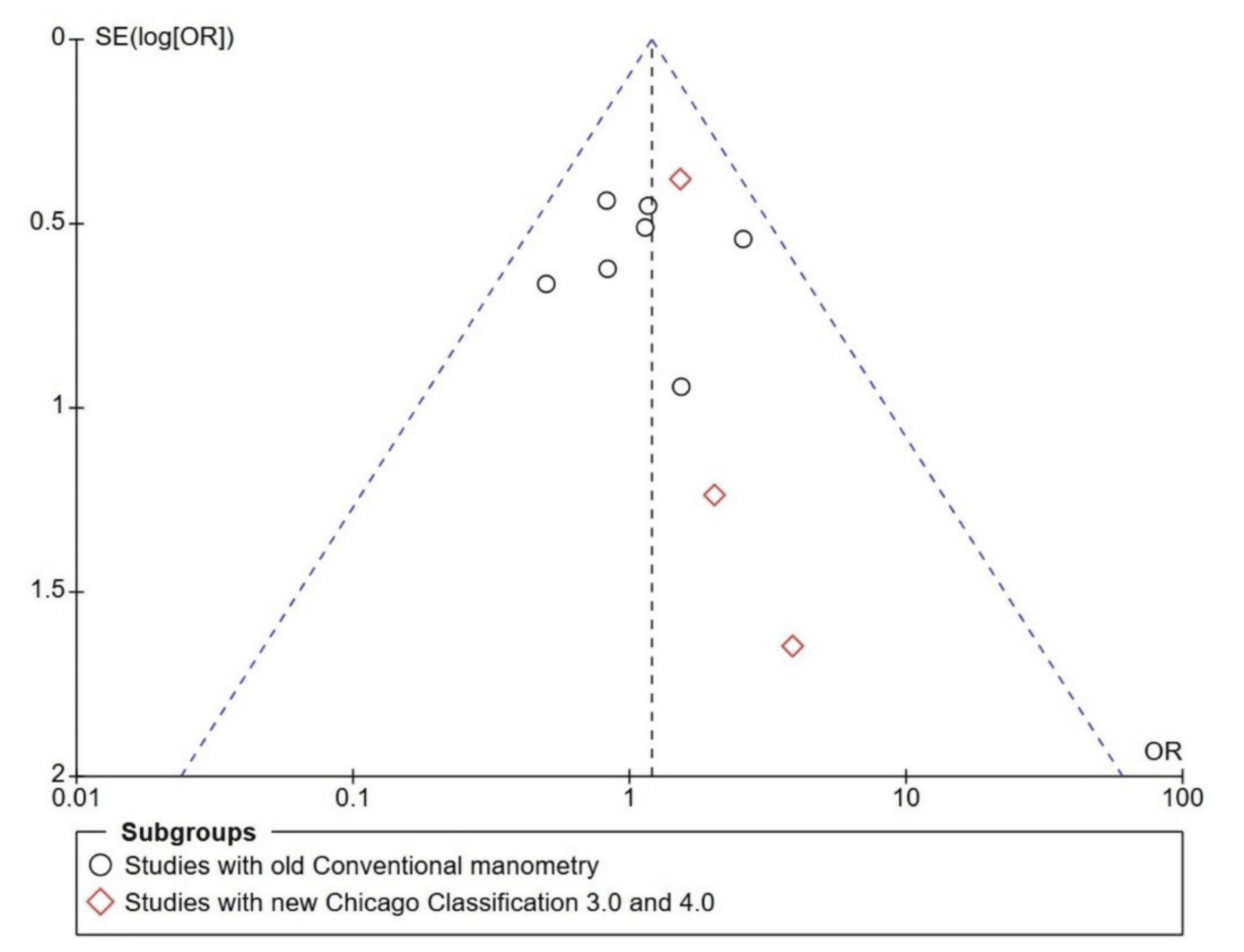
Funnel plot for publication bias considering analysis of studies with conventional manometry and HRM Black circles represent studies which considered conventional manometry [23-26, 28, 30, 32]. Red diamonds represent studies that considered high-resolution manometry [27, 29, 31].

The study's bias was analysed by two authors (VA and KA), and the opinion of a third author (SM) was sought if there was any doubt. The RoB for the randomised control trial was assessed using RoB 2 in RevMan 5.0. Figure 6 shows the RoB 2 Summary graph, and Figure 7 shows the RoB 2. For observational studies, the Newcastle-Ottawa Scale (NOS) score was calculated and is described in Table 4. A funnel plot for publication bias analysis was created using RevMan 5.0 and is represented in Figure 8. Figure 8 illustrates a Funnel plot comparing POD between IEM and non-IEM patients, suggesting no publication bias. However, the impact of smaller observational studies on the outcomes is also notable. Figure 9 shows a funnel plot comparing studies with conventional manometry and HRM, which also suggests there is no publication bias.

**Table 4:** Newcastle-Ottawa Scale (NOS) score for non-randomised studies.

Study	Selection score (4/4)	Comparability (2/2)	Outcome (3/3)	Total score (Max = 9)	Risk of Bias
Addo et al. [27]	****	*	***	8	Low
Booth et al. [28]	****	*	***	8	Low
Nikolic et al. [29]	****	*	***	8	Low
Ravi et al. [30]	****	*	***	8	Low
Munitiz et al. [31]	****	*	***	8	Low
Tsereteli et al. [32]	****	*	***	8	Low

Discussion

GERD is a common indication for fundoplication. Tailoring the different types of fundoplication for a patient with pre-existing IEM is a dilemma that is common among upper GI surgeons, who are concerned about worsening POD due to IEM. This widespread belief that NF causes worsening POD in patients who have pre-existing ineffective oesophageal motility is because of weak peristalsis against the 360-degree wrap. The reasons for postoperative dysphagia in Nissen fundoplication are a tight 360° wrap, long ramp peptic stricture oedema and inflammation, wrap torsion, unrecognised preoperative motility disorders like achalasia, hypercontractile oesophagus, and angulation of the wrap [33]. A tight NF, by the nature of the surgery itself, can lead to POD [34, 35]. For the same reason, TF can result in less POD as there is no tight wrap, and it is a partial wrap. Early achalasia cardia is another dreadful postoperative cause of dysphagia after Nissen fundoplication. However, this study suggests that there is no statistical significance indicating worsening postoperative dysphagia in IEM patients.

The incidence of IEM is approximately 20% among patients who undergo manometry [36], and more than 50% of symptomatic patients have IEM [37, 38]. The Chicago classification 4.0 is changing the criteria of ineffective oesophageal motility, as this is overdiagnosed. This indirectly suggests that IEM is a minor disorder, often overlooked as a preoperative finding, and therefore NF is not considered, despite NF and TF providing equal reflux control [39]. Lee et al., in their meta-analysis comparing all types of fundoplication, concluded that there is no difference in reflux control among the three significant types of fundoplication, with TF providing better POD [40]. The causes of ineffective oesophageal motility include medical conditions such as scleroderma and achalasia cardia. One cause of IEM is oesophageal inflammation. GERD causes distal oesophageal inflammation, and there is a high chance that GERD results in IEM. The authors think that if GERD is treated early, there is a chance the IEM can be reversed.

In this study, we tried to analyse the effects of IEM in patients following NF and identify the overall impact of NF on IEM patients. This study suggests that NF does not primarily affect pre-existing IEM, and the POD is not primarily due to IEM (P = 0.30). There is a high possibility that surgical techniques or other factors can influence the POD in a pre-existing IEM. We concluded that POD in IEM and non-IEM patients is not significant. The reason we compared preoperative and POD in IEM patients is to determine whether NF has improved symptomatic IEM. RCTs have shown no statistical significance, indicating no improvement in POD in IEM patients. However, observational studies have demonstrated that POD in IEM has improved. The study concluded that POD improved in patients with preoperative IEM (P=0.01).

The advantages of this study include filling a unique evidence gap in the literature. It will provide the foregut surgeons with more insight into the IEM and oesophageal hypomotility in patients who suffer from GERD. The advantages of this study lie in its unique comparison of IEM and non-IEM patients following NF. There is no meta-analysis to compare the effects of IEM on NF. Hajibandeh et al. compared the impact of oesophageal motility on NF and TF and concluded that the POD in TF is less compared to NF. However, the meta-analysis has not shown that NF worsens the POD in IEM patients [15]. Many individual observational and RCT studies, included in this meta-analysis, have demonstrated that the POD following NF is not significant. The other advantage of this study is that we have compared RCTs and observational studies as distinct subgroups, clearly indicating which study design has the most significant impact on the overall effects of the study. This study provides robust evidence for the IEM literature.

This study included patients with various definitions of IEM, as the definitions evolved with the introduction of HRM into practice. However, a subgroup analysis categorising studies by whether they used conventional manometry or HRM showed no statistical difference in POD between IEM and non-IEM patients. Interestingly, the subgroup analysis categorising studies by traditional and HRM approaches revealed an improvement in POD in IEM patients following NF, with a statistically significant p-value of 0.0004.

A limitation of the study is that observational studies dominate the overall effect. The patients included in the IEM had different definitions at different times. The definition of the IEM changed in the last decade. This is to avoid overdiagnosis of the IEM. This limitation was overcome by subgroup analysis of the studies, categorising those that used conventional manometry and those that used HRM. Although this is a limitation, it is also considered an advantage in the study because, as the definition changes, the POD is expected to remain high in patients with IEM, and the number of events is anticipated to increase. On the contrary, Figure 4 and Figure 5 illustrate that there is no difference in results between the conventional and high-resolution manometers when defining the IEM. The two RCTs by Zornig et al. [23] and Strate et al. [25] share many similarities, including mean age and mean BMI; however, the articles were published in different years. Moreover, the events and data are distinct, so RCTs were included in this study. Although the funnel plot shows an inverted funnel, events in the small studies skew the study's Odds ratios [29, 31, 32].

The results of this study demand careful consideration of its limitations to ensure a thorough and accurate interpretation. The secondary outcomes, such as reflux control, are not as well-concentrated, as all included studies reported reasonable postoperative reflux control. The new onset of dysphagia or de novo dysphagia following NF in both IEM and non-IEM groups has to be analysed as a separate study. In many of the included studies, the mean BMI was within normal range. So the results cannot be the same for the obese population. The study participants had associated HH, and the included studies did not account for HH in their analyses.

## Conclusions

In this study, there is sufficient evidence to suggest that NF improves POD in IEM patients. However, this evidence is from retrospective studies included in the analysis. The RCTs reveal no improvement in POD in IEM patients or worsening of POD in IEM patients following NF. Results from observational studies show improvement in POD in IEM patients, and overall effects indicate that POD in patients with pre-existing IEM following NF has improved pre-existing dysphagia in IEM patients, driven by heterogeneous, non-randomised data. There is no difference in POD between patients with IEM and those without IEM following NF. This result is from non-heterogeneous data from older studies. RCTs with better protocols can provide more insight into this ongoing debate about tailoring the type of fundoplication for IEM patients.
